# Bridge Points Guided Neural Motion Planning in Complex Environments with Narrow Passages

**DOI:** 10.3390/s26051582

**Published:** 2026-03-03

**Authors:** Songyi Dian, Juntong Liu, Guofei Xiang, Xingxing You

**Affiliations:** 1 Department of Automation, College of Electrical Engineering, Sichuan University, Chengdu 610065, China; 2 State Key Laboratory of Intelligent Construction and Healthy Operation and Maintenance of Deep Underground Engineering, Sichuan University, Chengdu 610065, China

**Keywords:** motion planning, narrow passages, bridge test, neural planner

## Abstract

Motion and path planning are fundamental to intelligent robotic systems, enabling navigation. The objective is to generate collision-free trajectories in obstacle-rich configuration spaces (C-spaces) while meeting performance constraints. In environments with narrow passages planning becomes especially difficult, as feasible regions have low measure and are rarely reached by random sampling. Classical sampling-based planners are probabilistically complete but inefficient in such regions. Learning-based planners like MPNet offer fast inference but often produce infeasible paths in cluttered areas, requiring expensive postprocessing. To address this trade-off, we propose a hybrid framework that combines improved sampling, structural abstraction, and neural prediction. A modified bridge-test sampler applies directional perturbations and corridor checks to generate reliable narrow passage samples. These are clustered into a sparse set of representative bridge points, which serve as nodes in a global graph. At query time, a greedy heuristic search explores this graph, using a neural local segment generator to connect nodes. We validate the approach on 2D maze maps, 3D voxel environments, and a 12-DOF manipulator performing a plugging task inside a simulated nuclear steam generator. Across all tasks, our method significantly outperforms classical and learning-based baselines in terms of success rate and planning time in narrow-passage-dominated scenarios. The inclusion of the repair module, under relaxed assumptions, also allows the framework to retain a generalized form of probabilistic completeness.

## 1. Introduction

Motion planning in environments with narrow passages remains a fundamental challenge in robotics because feasible solutions occupy a low-measure subset of the configuration space. Classical sampling-based planners such as Rapidly-exploring Random Trees (RRT) [1] and RRT* [2] explore the space through repeated random sampling and collision checking. They can be probabilistically complete under standard assumptions, yet their efficiency degrades sharply in cluttered scenes: most samples fall in irrelevant open regions and the probability of hitting passage entrances becomes extremely low. Bidirectional variants such as BiRRT [3] partially improve connectivity, but they still lack an explicit mechanism to concentrate computation on the critical bottleneck geometry.

In contrast, learning-based planners such as MPNet [4] leverage offline demonstrations to infer waypoints with low inference cost, effectively injecting a data-driven prior to speed up long-horizon planning. However, this prior can be brittle near tight gaps: under distribution shift, predicted waypoints may miss the correct passage entrances or violate constraints, which then triggers costly post-hoc repairs (e.g., RRT-Connect [3]) and offsets the gain of fast inference. This contrast highlights a key gap: sampling-based methods are guarantee-driven but budget-inefficient in narrow passages, whereas learning-based methods are fast but may fail to generalize at bottlenecks.

Therefore, our goal is not to replace geometric heuristics with learning, but to factorize the long-horizon planning problem into two complementary sub-problems: (i) topology exposure, where an explicit geometric mechanism extracts reliable bottleneck evidence (bridge points) to reveal passage structure; and (ii) fast local connection, where a learned segment generator amortizes the cost of repeatedly connecting nearby states once the topology is exposed. The bounded repair and backtracking mechanism serves as a safety net that preserves feasibility and robustness when the learned connector fails in tight regions.

In this paper, we propose a hybrid path planning framework that integrates geometric structure awareness with neural segment generation, achieving both speed and robustness. Our method first applies an improved bridge sampling strategy that perturbs candidate points along orthogonal directions and filters them using a single-channel criterion, effectively concentrating samples within true narrow passages while eliminating spurious points near obstacle boundaries or open space. The remaining valid bridge points are then clustered using DBSCAN to form a sparse set of representative passage centroids, which serve as global nodes in a sparse planning graph.

Between these nodes, a lightweight neural segment generator predicts feasible local trajectories. If the connection fails, a bounded number of RRT-Connect repairs are attempted, and backtracking is triggered if necessary to preserve completeness. Unlike traditional graph-based planners, our method avoids exhaustive graph construction or enumeration of node permutations, and instead employs a greedy heuristic guided by distance and residual goal cost, significantly reducing inference time while maintaining solution reliability.

We validate our method across three representative domains: (1) 2D maze environments with varying corridor layouts, (2) 3D spatial tasks involving double-hole and obstructed barriers, and (3) steam generator maintenance scenarios involving a 12-DOF robotic manipulator in a confined operational space. Experimental results demonstrate that our bridge-guided neural planner consistently outperforms classical sampling methods, bidirectional planners, and purely learned generators in success rate, planning time, and path quality. Furthermore, we provide a theoretical analysis showing that the overall planner inherits probabilistic completeness from the sampling-based repair component under standard assumptions, i.e., the success probability approaches one as the planning budget increases. The main contributions of this work are:

- Bridge-guided topology exposure with higher-quality bottleneck evidence. Compared with the classic Bridge Test and obstacle-based narrow-passage samplers, we propose an improved bridge sampling strategy (orthogonal perturbation and single-channel filtering) that produces sparser yet more accurate passage evidence, reducing false positives in open regions and near obstacle boundaries. This yields a compact set of valid bridge points that better reveals passage topology under the same sampling budget.
- Sparse structural abstraction for global guidance. Unlike using dense passage samples directly or constructing exhaustive graphs, we cluster valid bridge points via DBSCAN and use the resulting centroids as sparse global beacons to guide long-horizon planning. This abstraction reduces the number of global nodes and mitigates redundant exploration, enabling efficient planning without enumerating node permutations.
- Learning-accelerated local connection with completeness-preserving repair. In contrast to purely data-driven local planners that may fail under distribution shift, we integrate a lightweight neural segment generator with bounded RRT-Connect repair and backtracking. The learned connector amortizes repeated local connections once topology is exposed, while the sampling-based repair serves as a safety net that preserves feasibility and robustness in tight regions and supports probabilistic completeness under standard assumptions.

## 2. Related Works

Sampling-based motion planning methods such as RRT [1] and PRM [5] remain effective in high-dimensional spaces, yet narrow passages correspond to low-measure feasible sets and therefore dominate failure cases. We review related efforts from four complementary perspectives: (i) narrow-passage biased sampling, (ii) tree/roadmap expansion strategies, (iii) learning-based acceleration, and (iv) other complementary paradigms. Throughout this section, we emphasize how these lines of work motivate our bridge-guided topology exposure and completeness-preserving hybrid design.

### 2.1. Narrow-Passage Biased Sampling

These methods reshape the sampling distribution to increase the probability of entering bottlenecks. Representative ideas include expansiveness-driven exploration such as EST [6], bottleneck-aware sampling guided by geometric preprocessing or distance-field reasoning [7,8], and adaptive/informed sampling that concentrates effort in promising cost regions [9]. Bridge Test [10] is a classical technique that probes narrow regions by sampling “bridge” configurations across obstacles; it has inspired many subsequent variants. Other distribution-shaping strategies include dynamic-domain ideas [11], density/entropy-guided resampling [12,13], and boundary retraction [14]. Learning can also be used to bias sampling by learning connectivity structure or manifolds to improve coverage in bottlenecks [15]. Different from prior samplers that mainly increase the quantity of bottleneck samples, our improved bridge strategy aims to produce sparser but higher-quality passage evidence (lower redundancy and fewer spurious samples in open space), which is crucial for constructing a compact global guidance structure.

### 2.2. Tree/Roadmap Expansion and Bottleneck Connectivity

A complementary line improves connectivity by modifying how trees/roadmaps expand near constrained regions. Multi-tree or local-tree schemes can seed auxiliary trees around hard-to-connect samples and later merge them to improve passage traversal [16,17,18]. Other approaches compute local geometry to guide expansion directions, e.g., obstacle-aware steering [19] or PCA-based principal passage direction estimation [20,21]. More recently, multilevel planning isolates bottlenecks as low-dimensional sections and stitches solutions to boost success in high-DOF narrow passages [22]. Some works also incorporate bridge-test or directional heuristics into RRT-style expansion to improve entrance discovery [23]. In contrast, we do not rely on exhaustive multi-tree growth or expensive geometric computation at every expansion step. Instead, we explicitly expose passage topology via bridge evidence and then solve long-horizon planning on a sparse set of passage centroids, delegating only short-range connections to a lightweight local connector.

### 2.3. Learning-Based Planning Acceleration

Learning-based planners attempt to amortize planning cost by predicting paths or local connections from data. MPNet [4] learns a latent environment representation and predicts coarse trajectories quickly, but may fail under distribution shift or in very tight corridors where accurate entrance discovery is required. Other methods learn feasibility or guidance signals on discretized workspaces [24,25], or extract geometric features from point clouds (e.g., PointNet-style encoders) to model free-space structure [26]. Reinforcement learning has also been combined with sampling-based planners to learn extension policies in difficult environments [27]. Our method treats learning as a local accelerator rather than a standalone planner: once topology is exposed by geometric bridge evidence, a lightweight neural segment generator connects nearby states, and a sampling-based repair is invoked when learning fails in tight regions.

### 2.4. Other Complementary Paradigms

Optimization and decomposition-based approaches offer alternative perspectives on constrained planning, e.g., tightening collision constraints in a homotopy-based trajectory optimization framework [28], safe-box graph decomposition with convex smoothing for provably safe navigation [29], and enhanced variants combining goal biasing and smoothing for industrial narrow gaps [30]. Importance-sampling bidirectional planners also improve efficiency in specific domains such as micro-scale ducts  [31,32,33]. These works highlight the broader trend of combining global structure reasoning with fast local solvers, which aligns with our hybrid design that couples sparse passage-graph guidance with learning-driven local connection and completeness-preserving repair.

## 3. Methodology

**Figure 1:**
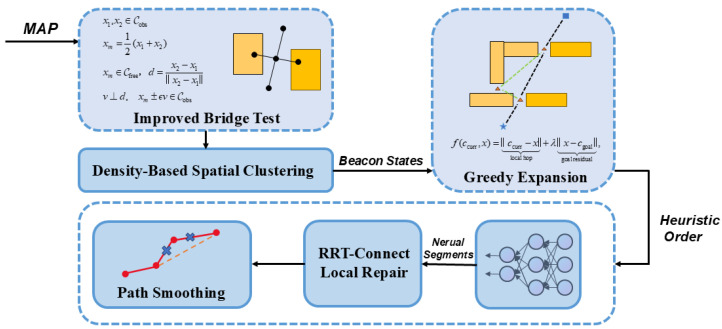
Bridge points guided neural motion planning pipeline. Given a map, Improved Bridge Testgenerates candidate configurations concentrated around narrow passages by orthogonal perturbation and validity checks. Density-Based Spatial Clustering (DBSCAN) groups valid candidates and outputs a sparse set of representative beacon states. Greedy Expansion orders and selects beacons to expose the long-horizon passage topology without exhaustive graph enumeration. A lightweight network predicts Neural Segments between successive beacons; if a predicted segment is infeasible, RRT-Connect Local Repair is invoked to recover feasibility. Finally, the concatenated path is refined by Path Smoothing to produce the final path.

This section details the three core components of our bridge-guided path-planning framework, whose overall workflow is illustrated in Figure 1. Section A explains how potential narrow-passage key points are extracted from the configuration space: an improved bridge-sampling procedure, followed by DBSCAN clustering, yields a sparse yet representative set of bridge points. Section B introduces the learning-driven local segment generator, which predicts collision-free trajectory segments between successive bridge points and validates them through fast collision checking, thereby populating the edge set of the inter-segment connection graph. Section C focuses on the query phase, where a greedy expansion strategy heuristically orders bridge points and iteratively invokes the local planner; any failed link is repaired by RRT-Connect, and the concatenated path is finally smoothed to obtain the end-to-end trajectory.

### 3.1. Bridge Point Sampling and Representative Extraction

**Figure 2:**
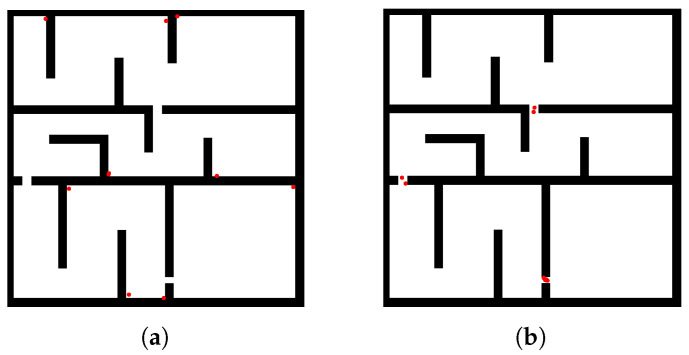
Comparison of bridge-point sampling behaviors in a narrow-passage scene. Each red dot denotes an accepted bridge midpoint after collision checks. (**a**) The classic Bridge Test tends to accept midpoints near concave corners, producing many false positives that do not represent the passage interior. (**b**) Our improved variant suppresses these corner samples and concentrates accepted points along the geometrical centerline of the true narrow corridor, yielding a cleaner and more informative bottleneck evidence set for subsequent clustering.

To efficiently identify potential narrow passage regions in the workspace, this paper proposes an improved bridge sampling strategy based on the traditional Bridge Test, applicable to both 2D and 3D spaces. The method begins by randomly sampling two points within obstacles. If the midpoint of the line segment connecting these two points lies in free space, it is regarded as a candidate bridge point. To filter out pseudo-bridge points located at open-space edges without meaningful passability, a directional constraint mechanism is introduced: local perturbation sampling is conducted along directions orthogonal to the original line segment. These perturbations are used to check for the presence of clear obstacle boundary features. A point is considered a valid bridge point only if there exists exactly one direction with free space among the orthogonal directions, indicating structural “single-channel traversability.” Figure 2 illustrates the effect: the classical Bridge Test (a) produces many spurious samples at concave corners, whereas the proposed test (b) confines accepted points to the true narrow-passage region.

Through multiple rounds of sampling and filtering, a high-quality set of candidate bridge points is obtained. Considering that the candidate bridge points may be densely distributed and redundant, the Density-Based Spatial Clustering of Applications with Noise (DBSCAN) algorithm is further employed to perform clustering on the candidate set. The centroid of each cluster is selected as the representative bridge point, resulting in a sparse, well-covered set of representative bridge points. This set serves as the core node structure of the subsequent path graph, laying the foundation for edge generation and path search.

As shown in Algorithm 1, the procedure consists of three stages: (1) sampling two collision points and testing their midpoint (Lines 4–6), (2) directional perturbation filtering to enforce single-channel traversability (Lines 7–17), and (3) DBSCAN-based clustering to extract representative centroids (Lines 20–21).
**Algorithm 1** Improved Bridge Sampling  1:**INPUT**: state bounds B, number of samples *N*, perturbation distance δ, clustering parameters ϵ,minPts  2:**OUTPUT**: representative centroids *C*  3:B←∅  4:**for** i=1 **to** *N* **do**  5:    sample two points x,p∼Uniform(B)  6:    let m←(x+p)/2  7:    **if** *m* is collision-free **then**  8:        compute orthonormal directions $\left\{d_{k}\right\}$  9:        let f←010:        **for** each $d_{k}$ **do**11:            let $m_{k}\leftarrow m+\delta \;d_{k}$12:            **if** $m_{k}$ is collision-free **then**13:                f←f+114:            **end if**15:        **end for**16:        **if** f=1 **then**17:           B←B∪{m}18:       **end if**19:    **end if**20:**end for**21:C←DBSCAN(B,ϵ,minPts)22:**return** *C*

### 3.2. Neural Local Segment Generation

Given the sparse beacon set *C* produced by Algorithm 1 (nodes of the planning graph), the planner constructs edges on demand by calling a local connector to generate feasible segments between selected beacon pairs. Concretely, Algorithm 2 implements $\mathrm{LocalConnect}(c_{i},c_{j})$, and Algorithm 3 uses greedy expansion to decide which $\left(c_{i},c_{j}\right)$ to connect next.
**Algorithm 2** Neural Local Segment Generator  1:**INPUT**: beacon pair $\left(c_{0},\;c_{\mathrm{goal}}\right)$, environment latent *Z*, neural model N, max steps *T*, threshold ε  2:**OUTPUT**: local segment $\tau (c_{0}\;\to \;c_{\mathrm{goal}})$ or failure  3:$c_{\mathrm{curr}}\leftarrow c_{0}$  4:**for** t←1 **to** *T* **do**  5:   $c_{\mathrm{next}}\leftarrow N\left(Z,\;c_{\mathrm{curr}},\;c_{\mathrm{goal}}\right)$  6:   **if** $∥c_{\mathrm{next}}-c_{\mathrm{goal}}∥\;\leq \;\varepsilon$ **then**  7:       append $c_{\mathrm{goal}}$ to τ  8:       **return** τ  9:   **end if**10:   $\sigma \leftarrow \mathrm{Interpolate}(c_{\mathrm{curr}},\;c_{\mathrm{next}})$11:   **if** CollFree(σ) **then**12:       append all points in σ to τ13:       $c_{\mathrm{curr}}\leftarrow c_{\mathrm{next}}$14:   **else**15:       $\rho \leftarrow \mathrm{RRTConnect}(c_{\mathrm{curr}},\;c_{\mathrm{next}})$16:       **if** ρ≠failure **then**17:          append all points in ρ to τ18:          $c_{\mathrm{curr}}\leftarrow c_{\mathrm{next}}$19:       **else**20:          **BACKTRACK**: remove last two points from τ21:          $c_{\mathrm{curr}}\leftarrow$ last point in τ22:       **end if**23:   **end if**24:**end for**25:**return** failure

**Algorithm 3** Bridge-Guided Hybrid Planner (Overall Pipeline)
  1:**INPUT**: start $c_{s}$, goal $c_{g}$, state bounds B, obstacle map $x_{\mathrm{obs}}$  2:**OUTPUT**: feasible path π or failure  3:

$Z\leftarrow \mathrm{EnvEncoder}(x_{\mathrm{obs}};\;\theta^{e})$

  4:C←**Algorithm 1**(B, N, δ, ϵ, minPts)                      // bridge beacons (nodes)  5:initialize open set $O\leftarrow \{c_{s}\}$, path $\pi \leftarrow [c_{s}]$  6:**while** O≠∅ and budget not exhausted **do**  7:     select next beacon $c_{\mathrm{next}}$ from *C* by greedy cost (distance + goal residual)  8:     τ←**Algorithm 2**$\left(c_{\mathrm{curr}},\;c_{\mathrm{next}},\;Z,\;N,\;T,\;\varepsilon \right)$  9:     **if** τ≠failure **then**10:          append τ to π, set $c_{\mathrm{curr}}\leftarrow c_{\mathrm{next}}$11:     **else**12:         backtrack / expand alternative beacon candidates13:     **end if**14:     **if** $∥c_{\mathrm{curr}}-c_{g}∥\;\leq \;\varepsilon$ **then**15:        **return** π16:     **end if**17:
**end while**
18:**return** failure


First, the environment map $x_{\mathrm{obs}}$ is encoded into a low-dimensional latent variable *Z* via the environment encoder:

(1) $$Z=\mathrm{EnvEncoder}\left(x_{\mathrm{obs}};\;\theta^{e}\right).$$

This latent *Z* captures multi-scale spatial features of the obstacle distribution through a multi-layer convolutional and pooling architecture.

Given the current configuration $c_{t}$ and target configuration $c_{\mathrm{goal}}$, the state predictor—a multi-layer perceptron (MLP) with ReLU activations—outputs the next-step configuration:

(2) $${\hat{c}}_{t+1}=\mathrm{StatePredictor}\left(Z,\;c_{t},\;c_{\mathrm{goal}};\;\theta^{p}\right).$$

During training, successful trajectories $\left\{c_{0}^{*},\;c_{1}^{*},\ldots ,c_{T}^{*}\right\}$ generated by ${\mathrm{RRT}}^{*}$ planners serve as demonstrations. A sliding window of length τ is applied to each demonstration trajectory to form transition samples. Each sample consists of

(3) $$\left(\;Z,\;c_{t}^{*},\;c_{\mathrm{goal}}^{*},\;\left\{c_{t-1}^{*}\right\}\right)$$

as inputs, with the next-step configuration $c_{t+1}^{*}$ as the supervised label.

The network parameters $\theta^{p}$ are learned by minimizing the mean squared error between predicted and true configurations:

(4) $$\ell_{\mathrm{path}}=\frac{1}{N_{p}}\sum_{j=1}^{N_{p}}\sum_{i=0}^{\tau -1}{∥\;{\hat{c}}_{j,i+1}^{*}-c_{j,i+1}^{*}∥}^{2}.$$

During inference, the model iteratively predicts ${\hat{c}}_{t+1}$ from $\left(Z,\;c_{t},\;c_{\mathrm{goal}}\right)$ until $∥c_{t+1}-c_{\mathrm{goal}}∥\;\leq \;\varepsilon$, where ε is a distance threshold. Upon reaching the vicinity of the goal, the generation stops, yielding a trajectory segment $\left\{c_{0},\ldots ,c_{T}\right\}$.

Each adjacent pair $\left(c_{i},\;c_{i+1}\right)$ is then validated: if a direct Steer-To connection is collision-free, it is kept; otherwise, RRT-Connect is invoked to repair that segment. By concatenating all successful partial segments, a globally feasible path from start to goal is efficiently constructed.

### 3.3. Greedy Expansion with a Distance–Goal Composite Cost

Once the bridge points have been clustered, the planner proceeds in a purely greedy fashion, but the candidate ordering is no longer determined by “nearest first” alone. Let $c_{\mathrm{curr}}$ be the current configuration, $c_{\mathrm{goal}}$ the goal, U⊆C the set of as-yet unvisited bridge centres, and S a LIFO stack that stores the committed waypoints (S=∅ at start-up). At every expansion cycle the planner queries the *k* geometrically closest points to $c_{\mathrm{curr}}$ in the set $U\cup \{c_{\mathrm{goal}}\}$, denoted $N=\{x_{1},\ldots ,x_{k}\}$. For each candidate x∈N, it evaluates the *composite cost*

(5) $$f\left(c_{\mathrm{curr}},\;x\right)=\underset{\mathrm{local}\;\mathrm{hop}}{\underbrace{∥c_{\mathrm{curr}}-x∥}}+\lambda \;\underset{\mathrm{goal}\;\mathrm{residual}}{\underbrace{∥x-c_{\mathrm{goal}}∥}},$$

where the weight λ∈[0, 1] biases how eagerly the search moves towards the goal (λ=0 yields the original “pure nearest” rule; λ≈0.3 proved robust in our experiments). The candidates are sorted in ascending *f*; the planner then tests them in that order, invoking the local segment generator up to $R_{\max}$ times per candidate (each call limited by a run-time budget $T_{\max}$). The first candidate that returns a collision-free path is accepted: the segment is spliced into the global solution, $c_{\mathrm{curr}}$ is updated to the newly reached point, that point is removed from *U*, and it is pushed onto S. If every candidate fails, the algorithm backtracks one step by popping S; exhaustion of the stack signals overall failure. Conversely, as soon as $c_{\mathrm{curr}}$ links directly to $c_{\mathrm{goal}}$, the concatenated stack constitutes a complete solution path. Compared with the pure-nearest scheme, this composite-cost heuristic keeps the search lightweight while sharply reducing the likelihood of wandering into dead-end corridors, yielding millisecond-level expansions in all tested scenarios.

### 3.4. Probabilistic Completeness

The proposed planner is probabilistically complete, i.e., if there exists a collision-free path in the configuration space $C_{\mathrm{free}}$, then as the number of bridge-sampling iterations N→∞ and the number of local repair tries per segment $R_{\max}\to \infty$, the probability that the algorithm finds a solution approaches one.

Let $\pi^{*}$ be any collision-free path from the start $q_{\mathrm{start}}$ to the goal $q_{\mathrm{goal}}$. Because $C_{\mathrm{free}}$ is an open set, there exists a finite sequence of waypoints

(6) $$q_{\mathrm{start}}=w_{0},\;w_{1},\;\ldots ,\;w_{K}=q_{\mathrm{goal}}$$

such that straight-line connections between consecutive $w_{i}$ lie entirely in $C_{\mathrm{free}}$. We will show that the planner will almost surely discover a sequence of bridge-points clustered near these waypoints and successfully connect them.

- Sampling of Bridge Points.Each bridge-sampling iteration samples two collision configurations in $C_{\mathrm{obs}}$ and retains their midpoint *m* if it lies in $C_{\mathrm{free}}$ and passes the directional check. As N→∞, the standard Bridge Test is known to generate points whose density converges to that of the medial axis of $C_{\mathrm{free}}$. The added directional-filter retains only those midpoints lying in narrow passages, but does not prevent sampling arbitrarily close to any given narrow passage segment. Hence for each subsegment $\left[w_{i-1},\;w_{i}\right]$, there is a nonzero probability that some sampled midpoint falls within an ϵ-ball around it. By the law of large numbers, with probability one, every ϵ-ball around each $w_{i}$ will eventually contain at least one bridge point.
- Density-Based Clustering. DBSCAN with fixed ϵ and minPts clusters these midpoints. Since infinitely many samples appear in each ϵ-neighborhood of $w_{i}$, each neighborhood forms a cluster whose centroid lies within distance ϵ of $w_{i}$.
- Local Repair Completeness. Between each pair of neighboring centroids $c_{i-1}$ and $c_{i}$, we invoke up to $R_{\max}$ attempts of the local segment generator, falling back on RRT-Connect if needed. RRT-Connect itself is probabilistically complete: as the number of samples (here local repair attempts) goes to infinity, it will find a collision-free connection between any two points in the same connected component of $C_{\mathrm{free}}$ with probability one. Therefore, for sufficiently large $R_{\max}$, the probability of failing to join $c_{i-1}$ to $c_{i}$ tends to zero.

Combining these facts, for any fixed finite sequence of waypoints along $\pi^{*}$, the probability that (i) each vicinity of $w_{i}$ contains a bridge-cluster centroid and (ii) each pair of consecutive centroids is successfully connected tends to one as $N,\;R_{\max}\to \infty$. Hence the overall probability that the planner returns a complete collision-free path approaches one.

## 4. Experimental Results

### 4.1. Implementation Details

This work is implemented in MATLAB R2024a (MathWorks, Natick, MA, USA) using the Deep Learning Toolbox. All experiments are conducted on a workstation with an Intel i5-13400 CPU (Intel, Santa Clara, CA, USA), an NVIDIA RTX 4070 GPU, 32GB RAM (Nvidia, Santa Clara, CA, USA), and Windows 10 operating system.

Neural Network Architecture: We design a 4-layer multilayer perceptron (MLP) as the segment generator. The network input includes the current configuration, the goal configuration, and an environment encoding, while the output is a fixed-length trajectory segment. For 2D tasks, the environment is encoded as a flattened occupancy grid directly fed into the MLP. For 3D tasks, we use a convolutional neural network (CNN) to extract spatial features from voxel maps, which are then concatenated to the planner input.

Training Data: Separate datasets are constructed for different task domains:

- 2D navigation: We generate 500 maze-like maps with narrow passages (each of size 100×100), and sample 200 random start-goal pairs per map. Paths are computed using RRT*, yielding 100,000 total training trajectories.
- 3D spatial planning: We construct 10 voxel environments (5m×5m×2.5m) with double-hole walls and central obstacles. Each map provides 1000 RRT*-planned paths, totaling 10,000 trajectories.

Each of the above datasets is used to train a separate model. All test-time queries use unseen start-goal pairs to evaluate generalization.

Bridge Point Generation: We apply centroid-perturbed bridge sampling in free space. For each candidate obstacle pair, midpoints are sampled and validated via local collision checks to retain only those between real obstacle gaps. Valid bridge points are then projected back to high-dimensional configuration space using inverse kinematics and further filtered by reachability and collision constraints. Finally, DBSCAN clustering is used (with radius 3–5% of environment scale and minimum samples of 4) to extract centroid nodes for sparse graph construction.

Evaluation Settings: All planners are tested under a unified evaluation framework. Sampling-based methods are capped at 10,000 iterations. Learning-based planners are allowed to generate up to 150 neural proposals, and each RRT-Connect repair attempt is bounded by 5000 iterations.

### 4.2. Results

To evaluate the adaptability and effectiveness of the proposed path planning framework, we conduct comprehensive experiments across three representative task domains: 2D navigation, 3D volumetric motion, and high-DOF robotic manipulation. All methods are executed on a unified hardware platform under equal computational budgets.

**Figure 3:**
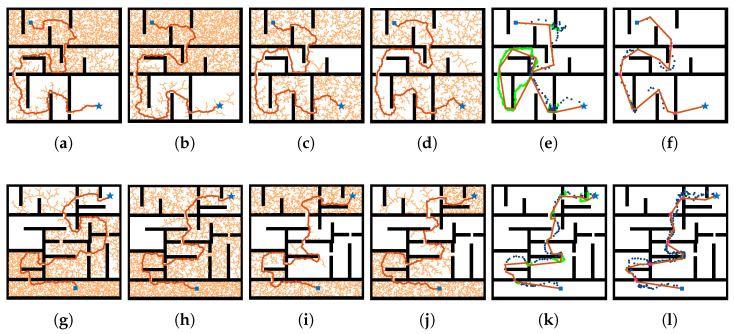
Qualitative comparison of six planners on two cluttered 2D workspaces. From left to right in each row: RRT, RRT*, BiRRT, RRT-Connect, MPNet, and the proposed method. (**a**–**f**) correspond to Map1; (**g**–**l**) to Map2. All planners were given the same computation budget. In the subfigures of MPNet and the proposed method, The blue square and star denote the start and goal configurations, the blue points denote the candidate outputs of the neural local segment generator, the green points represent the samples generated by the traditional planner, the triangles indicate the clustered bridge points, and the orange lines depict the final planned path.

**Figure 4:**
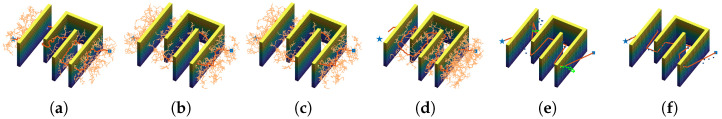
Qualitative comparison of six planners on the 3D workspace. From left to right: (**a**) BiRRT, (**b**) RRT-Connect, (**c**) RRT*, (**d**) RRT, (**e**) MPNet, (**f**) proposed method. All planners share the same start and goal configurations and identical computation budgets.

**Figure 5:**
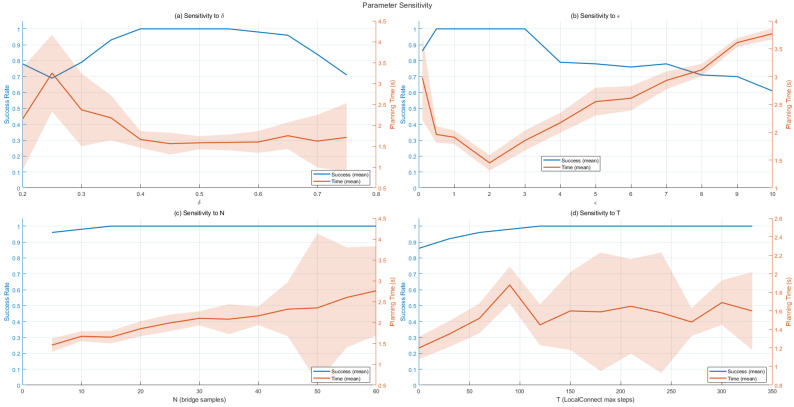
Parameter sensitivity on a representative 2D map. We vary the bridge perturbation distance δ, DBSCAN neighborhood radius ϵ, the number of bridge trials *N*, and the maximum rollout steps *T* in the local connector. The curves report success rate and planning time, where the orange shaded bands indicate ±1 standard deviation over repeated runs.

(1) 2D Navigation Tasks: We consider two types of randomly generated maze-like maps (Map1 and Map2), as shown in Figure 3, each sized at 100×100 grid cells, containing 3 and 6 narrow corridors, respectively. In Map1, although all planners succeed in some trials, sampling-based methods suffer from low success rates due to inefficient sampling in narrow regions (e.g., RRT achieves only 6.67%). MPNet demonstrates relatively shorter path lengths and lower planning times due to prior learning but struggles in Map2, where the increased number of narrow corridors causes predicted waypoints to miss passage entrances, leading to frequent fallback to classical planners. This significantly increases planning time, as observed in Map1 where MPNet produces a sample that crosses through a wall, necessitating full replanning. In contrast, our method traverses open regions efficiently while explicitly targeting passage entrances using bridge-point clusters. Under the tested budget and trial set, it solves all instances in both Map1 and Map2 (100% success), while also achieving the shortest average path length and the lowest planning time.

(2) 3D Navigation Tasks: As shown in Table 1 and Figure 4, in the constructed 3D environment characterized by dual narrow holes and dual barrier constraints, traditional sampling-based planners exhibit significant limitations in feasibility and efficiency. The irregular distribution of free space and the presence of severely constrained passage geometries hinder effective exploration, leading to frequent planning failures or suboptimal paths, especially for methods that rely on random expansion without structural awareness. Although learning-based approaches such as MPNet improve overall feasibility, they tend to rely heavily on fallback classical planners when encountering bottleneck regions—particularly around narrow holes—resulting in longer planning times and unnecessarily convoluted trajectories. This fallback mechanism highlights the difficulty of generalizing across tight passages using purely data-driven strategies. In contrast, our method demonstrates superior adaptability and efficiency in such constrained topologies. By explicitly modeling passage geometry, the planner is able to rapidly identify and traverse critical narrow regions. This structure-aware guidance not only ensures full task completion but also results in shorter planning times and lower trajectory costs with minimal variance. The proposed approach thereby achieves a favorable balance between success rate, path optimality, and computational efficiency in highly constrained 3D planning scenarios. We note that the 100% success on Map3D is an empirical statistic over the tested instances under a fixed budget; it indicates that our topology-aware guidance and completeness-preserving repair substantially improve entrance discovery and connectivity in the presence of multiple tight holes.

(3) Parameter Sensitivity: We conduct a controlled study on a representative 2D map by varying four key hyper-parameters in our framework: the bridge perturbation distance δ, the DBSCAN neighborhood radius ϵ, the number of bridge sampling trials *N*, and the maximum rollout steps *T* in the neural local connector. Figure 5 reports the success rate and planning time (mean ± standard deviation over repeated runs) under different parameter settings.

The perturbation distance δ governs the single-channel filtering in the improved bridge sampling (Algorithm 1). When δ is too small, the perturbations fail to sufficiently probe the local passage geometry, leading to less reliable bottleneck evidence and decreased success rates; when δ is too large, the perturbations may step outside the true corridor thickness and reject valid midpoints, which also degrades performance. As shown in Figure 5a, the best trade-off is achieved around δ=0.5, where the planner attains consistently high success while keeping the planning time low.

The parameter ϵ controls the clustering granularity of candidate bridge points. A very small ϵ tends to fragment a corridor into many small clusters, increasing the number of beacons and local connections, thus raising runtime; conversely, overly large ϵ may merge distinct bottlenecks and produce an over-sparse abstraction, weakening guidance and reducing success. Figure 5b suggests that ϵ∈[0.5, 1] yields the most stable behavior, balancing sparsity and representativeness of the passage centroids.

The number of bridge trials *N* allocates computation to topology exposure. Increasing *N* generally improves the probability of discovering passage entrances, but also increases sampling/validation cost. In Figure 5c, success saturates quickly as *N* increases, while the planning time grows with larger *N*. Therefore, we set N=20 as a practical knee point that preserves near-saturated success with moderate runtime overhead.

The rollout limit *T* bounds the horizon of the neural local segment generator (Algorithm 2). A small *T* may prematurely stop the connector before reaching the next beacon, causing additional repairs or failures; larger *T* increases the worst-case inference and checking cost. As shown in Figure 5d, T=150 provides a robust success-time trade-off. Notably, our connector terminates early once a feasible connection is found (i.e., the rollout stops when the goal vicinity is reached), so increasing *T* beyond this value has marginal impact on the final performance in practice.

Based on the above sensitivity analysis, we use δ=0.5, ϵ∈[0.5, 1], N=20, and T=150 as default hyper-parameters in the remaining experiments, as they provide a stable balance between success rate and planning time on the evaluated 2D tasks.

## 5. Application Case: Steam Generator Plugging Task

### 5.1. Task Description

**Figure 6:**
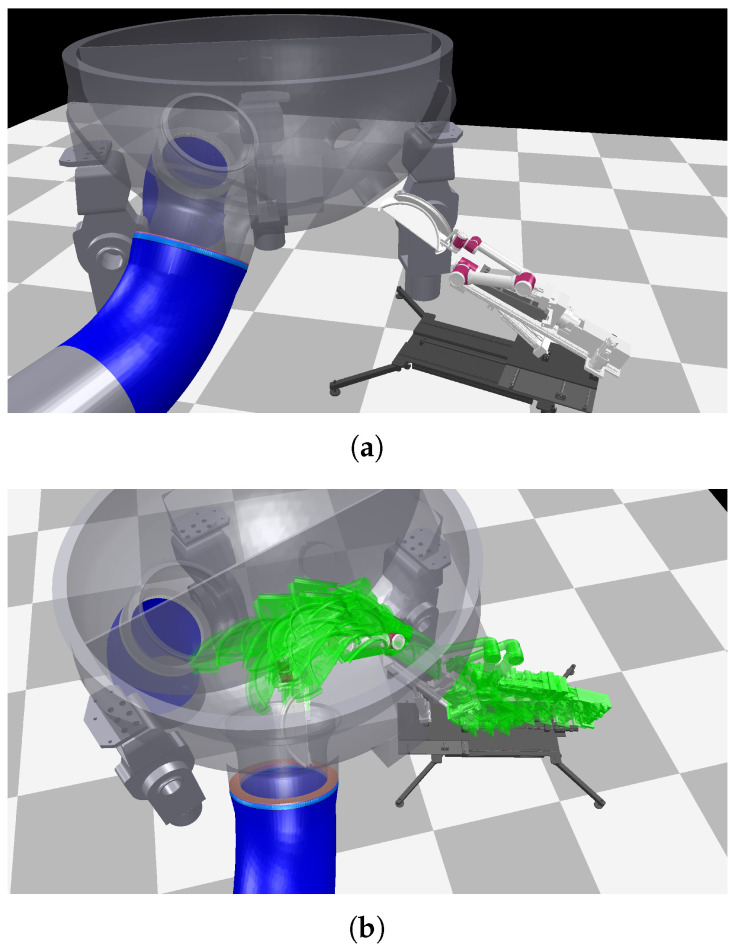
Demonstration in the steam-generator maintenance scenario. (**a**) Photorealistic layout of the steam generator with the 12-DOF plugging robot poised at the manway entrance. (**b**) Time-lapse rendering of the trajectory generated by the proposed bridge-guided planner.

The plugging task takes place inside the steam generator of a nuclear power plant, which is a sealed cylindrical vessel with complex internal structures and severely limited working space. The interior is only accessible through a narrow maintenance port. As shown in Figure 6, during scheduled maintenance, a 12-degree-of-freedom robotic manipulator is required to carry a plugging device through the manhole, navigate past the water chamber section, and reach the main pipe region. The plugging operation is executed precisely at a position 200 mm in front of the pipe opening, ensuring the isolation of the steam generator from the primary reactor loop. Throughout this process, the robot must satisfy strict joint limit and collision avoidance constraints. The task constitutes a high-dimensional motion planning problem with pronounced spatial bottlenecks. In such constrained environments, conventional sampling-based planners often fail to return feasible solutions within a reasonable time budget.

### 5.2. Training Data

To support this application, we construct a specialized dataset reflecting the spatial and kinematic constraints of the real deployment scenario. Specifically, we simulate a confined cylindrical workspace resembling the interior of a nuclear steam generator, incorporating narrow access ports and curved wall boundaries. Within this setup, we randomly sample 2000 start-goal configurations of the 12-DOF robotic manipulator. For each configuration, a reference trajectory is generated using RRT*, forming a domain-specific training dataset designed to capture the complexity of constrained insertion and alignment maneuvers.

### 5.3. Evaluation

We assess planner performance in the highly constrained steam-generator environment, which imposes tight pose and orientation requirements on a 12-DOF robotic arm. Quantitative results are summarized in Table 2. Under the fixed time budget used in our evaluation, classical sampling-based planners (RRT, RRT*, BiRRT, RRT-Connect) fail to return a complete feasible solution, which reflects the long-horizon difficulty of exploring a high-dimensional narrow-topology space under runtime constraints (rather than the absence of feasible solutions in principle). MPNet achieves a success rate of 68.6% and a mean path length of 7.67 m. However, it frequently violates goal tolerances or collision constraints near the bottleneck, which triggers repeated replanning/repair and leads to a long average runtime of 243 s. In contrast, our bridge-guided framework improves the success rate to 80.6% and reduces the mean path length to 7.09 m, while achieving an average runtime of 210 s. We note that the gain in runtime and path length over MPNet is moderate in this application case. This is expected because (i) our topology-exposure stage performs additional collision/IK validation for each bridge-test candidate, whose overhead partially offsets the benefit of faster neural local connections; and (ii) when MPNet succeeds, its repaired solution is already close to feasible, leaving limited room for further shortening the final path. Importantly, the main benefit of our method in this confined 12-DOF setting is improved robustness at the narrow-passage entrance and near the goal constraints, which reduces the frequency of costly replanning loops and yields a higher success rate under the same budget. These results suggest that while bridge-guided topology exposure remains effective in task-specific, high-DOF settings, further efficiency gains may require incorporating task-aware heuristics or learned IK-feasibility priors to reduce validation overhead.

### 5.4. Scalability to High-DOF Systems

As DOF increases, the dominant cost typically comes from geometric validation (collision checking and IK feasibility) rather than neural inference. Our method mitigates wasted exploration by factorizing long-horizon planning into (i) bridge-guided topology exposure that concentrates samples on bottleneck evidence and yields a sparse set of passage beacons (via DBSCAN), and (ii) fast local connections generated by a lightweight neural segment model. When the learned connector fails in tight regions, bounded RRT-Connect backtracking serves as a safety net, preserving feasibility and preventing catastrophic failures. Therefore, the scalability is mainly limited by the number of validation queries (bridge filtering and segment checking) times the per-query collision/IK cost, which grows with DOF and environment complexity. In future work, we will reduce this overhead by incorporating task-aware heuristics or learned IK-feasibility priors and by adopting hierarchical planning/refinement for very high-DOF systems.

## 6. Conclusions

This paper introduced a bridge-guided hybrid planner that combines an improved Bridge Test, DBSCAN-based clustering, and a learning-driven local planner to address motion planning for high-DOF robots in narrow passages. The proposed orthogonal-perturbation Bridge Test yields a sparser and more accurate set of passage samples, while the neural local planner backed by RRT-Connect repair achieves probabilistic completeness and competitive speed. Experiments on 2D and 3D benchmarks, as well as a 12-DOF steam generator scenario, demonstrated an 80.6% success rate and a 7.6% reduction in path length over MPNet, although the time advantage is partly offset by inverse-kinematics (IK) checks during bridge sampling, especially in sparsely obstructed environments.

## Figures and Tables

**Table 1:** Comparison of planners.

Maps	Algorithm	Time (s)	Path Length	Success Rate
Map1	RRT	6.71±0.57	326.08±7.25	6.67%
	RRT*	11.44±0.51	307.80±30.01	20.00%
	BiRRT	2.83±0.62	315.11±24.85	53.33%
	RRT-Connect	4.08±0.61	334.52±31.12	46.67%
	MPNet	3.75±0.57	276.31±14.79	73.33%
	Ours	1.58±0.16	240.51±8.26	100.00%
Map2	RRT	4.48±0.80	452.15±35.10	3.33%
	RRT*	10.10±1.10	441.30±28.50	16.67%
	BiRRT	3.58±0.75	470.22±40.12	33.33%
	RRT-Connect	4.35±0.90	463.50±36.00	40.00%
	MPNet	3.10±0.65	350.44±20.30	83.33%
	Ours	2.15±0.20	345.10±15.80	100.00%
Map3D	RRT	5.38±0.95	23.85±2.11	23.33%
	RRT*	20.21±1.20	22.20±1.72	40.00%
	BiRRT	4.77±0.88	25.28±2.26	53.33%
	RRT-Connect	6.50±0.92	26.51±2.14	50.00%
	MPNet	3.85±0.70	19.04±1.28	93.33%
	Ours	3.20±0.30	18.83±1.14	100.00%

**Table 2:** Results in the Steam Generator Scenario.

Algorithm	Time (s)	Path Length	Success Rate
RRT	–	–	0.00%
RRT*	–	–	0.00%
BiRRT	–	–	0.00%
RRT-Connect	–	–	0.00%
MPNet	243.24	7.67	70.00%
Ours	210.35	7.09	86.67%

## Data Availability

Data are available from the corresponding author upon reasonable request.
